# Are Genetic and Environmental Risk Factors for Psychopathology Amplified in Children with Below-Average Intelligence? A Population-Based Twin Study

**DOI:** 10.1007/s10519-023-10174-7

**Published:** 2024-02-14

**Authors:** Susanne Bruins, Elsje van Bergen, Maurits W. Masselink, Stefania A. Barzeva, Catharina A. Hartman, Roy Otten, Nanda N. J. Rommelse, Conor V. Dolan, Dorret I. Boomsma

**Affiliations:** 1 Department of Biological Psychology, Vrije Universiteit Amsterdam, Amsterdam, The Netherlands; 2 Amsterdam Public Health Research Institute, Amsterdam, The Netherlands; 3 Research Institute LEARN!, Vrije Universiteit Amsterdam, Amsterdam, The Netherlands; 4 Behavioural Science Institute, Radboud University, Nijmegen, The Netherlands; 5 Department of Psychiatry, Radboud University Medical Center, Nijmegen, The Netherlands; 6 Department of Psychiatry, University of Groningen, University Medical Center Groningen, Groningen, The Netherlands; 7 Karakter Child and Adolescent Psychiatry University Center, Nijmegen, The Netherlands; 8 Amsterdam Research and Development (AR&D) Research Institute, Amsterdam, The Netherlands

**Keywords:** Childhood psychopathology, Developmental psychology, Gene-environment interaction, Intelligence

## Abstract

There is a negative association between intelligence and psychopathology. We analyzed data on intelligence and psychopathology to assess this association in seven-year-old Dutch twin pairs (ranging from 616 to 14,150 depending on the phenotype) and estimated the degree to which genetic and environmental factors common to intelligence and psychopathology explain the association. Secondly, we examined whether genetic and environmental effects on psychopathology are moderated by intelligence. We found that intelligence, as assessed by psychometric IQ tests, correlated negatively with childhood psychopathology, as assessed by the DSM-oriented scales of the Child Behavior Check List (CBCL). The correlations ranged between − .09 and − .15 and were mainly explained by common genetic factors. Intelligence moderated genetic and environmental effects on anxiety and negative affect, but not those on ADHD, ODD, and autism. The heritability of anxiety and negative affect was greatest in individuals with below-average intelligence. We discuss mechanisms through which this effect could arise, and we end with some recommendations for future research.

**Supplementary Information:**

The online version contains supplementary material available at 10.1007/s10519-023-10174-7.

## Introduction

Intelligence is negatively correlated with psychopathology (Dietz et al. 1997; Pettersson et al. 2021). Thus, individuals with below-average intelligence are at greater risk for psychopathology. In the Netherlands, approximately 1 in 17 people are estimated to have borderline intellectual functioning or lower (IQ test score of 85 or lower), and 30 to 50 percent of these individuals suffers from psychopathology (Došen 2014). This pertains to between 300,000 and 500,000 individuals in the Netherlands, who have below average intellectual functioning and psychopathology. In this paper, we study the nature of the relationship between childhood psychopathology and intelligence from a genetic perspective: We ask how genetic and environmental sources of influence contribute to the negative correlation between intelligence and psychopathology, and whether genetic and environmental risks for psychopathology differ over the range of intelligence.

There are various mechanisms which may explain the negative relationship between intelligence and psychopathology. First, the system integrity theory states that individual differences in intelligence and psychopathology are a consequence of individual differences in the efficiency of complex physiological systems (Deary 2012). From the system integrity perspective, both lower intelligence and psychopathology symptoms are consequences of suboptimal functioning physiological systems. For example, disturbances in brain function may cause disturbances in cognitive processes such as executive functioning, which in turn could lead to symptoms of psychopathology and learning difficulties. There is some support for the system integrity theory of intelligence and psychopathology. Based on a combination of cognition-related measures as an indicator for system integrity, Caspi et al. found that individuals with more psychopathology symptoms show less system integrity (Caspi et al. 2014).

Second, the cognitive reserve hypothesis states that higher intelligence may function as a buffer for pathology. As such, higher intelligence is supposed to protect against risk factors for psychopathology (Koenen et al. 2009). For example, in stressful situations, individuals with average or above-average intelligence may find it easier to apply emotion-focused coping strategies, that is, to regulate their own emotions. These strategies require complex metacognitive skills. From studies on intellectual disability, we know that people with an IQ between 55 and 70 tend to cope with stressful social interactions by trying to change the situation itself, rather than their own response to the situation (Hartley and MacLean 2008). While problem-focus coping skills can be effective in situations where the situation can be altered, emotion-focused coping skills are more effective when the situation cannot easily be changed. Such situations may therefore have greater negative impact on individuals with lower intelligence.

Third, there is evidence that individuals with below-average intelligence are at increased risk for exposure to adverse experiences in childhood (Hassiotis et al. 2019). This implies that children with below-average intelligence have a greater environmental risk for psychopathology than their peers with average or above-average intelligence. For example, children with intellectual disabilities are at greater risk for social exclusion (Fisher et al. 2012; Schoop-Kasteler et al. 2022), to become victims of bullying (Christensen et al. 2012; McHugh and Howard 2017), to suffer sexual abuse (Stobbe et al. 2021), and to suffer exploitation and victimization in general (American Psychiatric Association 2013).

Finally, there may also be reciprocal effects of psychopathology on intelligence. Psychopathology may interfere with learning and with cognitive functioning. For example, both internalizing and externalizing psychopathology at ages 2 (Bub et al. 2007) and 7 (Zhou et al. 2010; Bodovski and Youn 2011) prospectively predict lower cognitive abilities and lower GPA later in childhood.

**Fig. 1:**
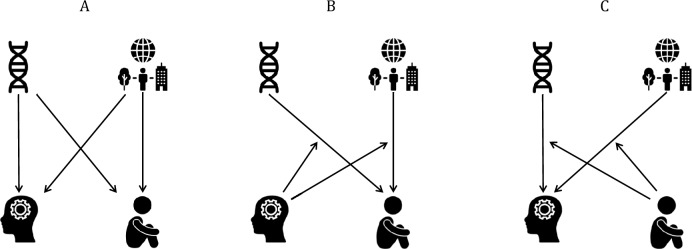
Mechanisms through which individuals with below-average intelligence might be at higher risk for psychopathology. **A**: Common genetic and environmental factors influence intelligence and psychopathology. **B**: Intelligence moderates genetic and environmental effects on psychopathology. **C**: Psychopathology moderates genetic and environmental effects on intelligence. Image credits. Figure 1 is created with images from Noun Project (https://thenounproject.com/). We thank Noun Project and the creators of these images: *DNA* by Warunk Icon from Noun Project (https://thenounproject.com/icon/dna-3500820/); *world environment* by Vector Portal from Noun Project (https://thenounproject.com/icon/child-sitting-on-the-floor-1578807/); *Child Sitting on the Floor* by Gan Khoon Lay from Noun Project (https://thenounproject.com/icon/child-sitting-on-the-floor-1578807/); *Brain* by Muhammad Taifik Sidik from Noun Project (https://thenounproject.com/icon/brain-4793958/)

In behavioral genetic studies, we decompose phenotypic variance into genetic sources of variance and environmental sources of variance, without necessarily identifying specific risk factors. From this perspective, there are various mechanisms that can drive the correlation between intelligence and psychopathology. First, there might be shared genetic and environmental factors that influence both intelligence and psychopathology (Fig. 1A). If intelligence is causally linked to psychopathology, this would also present as common genetic and/or environmental factors (in a multivariate twin model). Several studies in genetically informative designs have found negative genetic correlations (ranging from − 0.19 to − 0.38) and environmental correlations (ranging from − 0.17 to − 0.39) between intelligence and psychopathology (Jacobs et al. 2002; Grotzinger et al. 2019; Harden et al. 2020). Second, intelligence may moderate the effect of genetic and environmental influences on psychopathology: The genetic and environmental factors that confer risk for psychopathology are the same across the IQ range but their effects increase with decreasing IQ (Fig. 1B). Third, psychopathology may moderate intelligence (Fig. 1C). We note that these mechanisms are not mutually exclusive.

We examined the relationship and its etiology between psychometric IQ and five dimensions of psychopathology in 7-year-old twins. as assessed by the DSM-oriented scales of the CBCL. The five dimensions are (1) negative affect, which includes depressive symptoms and withdrawn behavior; (2) anxiety, which consists of anxiety- and phobia-related symptoms; (3) disobedient and defiant behavior (ODD); (4) autism, referring to problems with communication, affect, and flexibility; and (5) Attention Deficit Hyperactivity Disorder (ADHD), which includes attention problems, hyperactivity, and impulsive behavior. We estimated genetic and environmental correlations between these five indices of psychopathology and intelligence (Fig. 1A) in bivariate twin data, modeling the negative correlations between genetic and environmental effects on intelligence and psychopathology. Subsequently, we incorporated intelligence as a moderating factor in the relationship between genetic and environmental impacts on the five domains of psychopathology (Fig. 1B), and vice versa (Fig. 1C).

## Methods

This study was preregistered at https://doi.org/10.17605/OSF.IO/4DEGN. We outline the deviations from the pre-registered plan at the end of the Methods section.

### Participants

The data were collected from Dutch twins by the Young Netherlands Twin Register (YNTR; Bartels et al. 2007; Van Beijsterveldt et al. 2013; Ligthart et al. 2019). The YNTR recruits twins at birth (starting with the 1986 birth cohort), and follows them into adulthood. Parents of twins receive surveys about the twins’ development and behavior every few years. Here, we analyze maternally reported psychopathology symptoms at age seven.

In addition to developmental and behavioral surveys, there have been several longitudinal studies in which intelligence was assessed in a randomly selected subsample of YNTR participants (for a summary of the intelligence data and studies, see Franić et al. 2014). There were 28,239 twins with psychopathology data (14,089 complete twin pairs of which 5,168 MZ and 8,921 DZ pairs, and 61 incomplete twin pairs). For 1,089 twins there were data on psychopathology and intelligence (543 complete twin pairs of which 262 MZ and 281 DZ pairs, and 3 incomplete twin pairs). There were 148 twins with IQ data but no psychopathology data (73 complete twin pairs of which 23 MZ and 50 DZ pairs, and 2 incomplete pairs).

### Measures

Indices of psychopathology were based on the CBCL (Achenbach 1999; Achenbach et al. 2003). The CBCL is commonly used in research on mental health in children with more severe intellectual disability, and accurately reflects clinical psychopathology symptoms across the range of intelligence (Glasson et al. 2020). The CBCL consists of 112 items that are scored on a 3-point scale (0 = not true, 1 = somewhat or sometimes true, 2 = very true or often true). Achenbach developed DSM-oriented scales from experts’ ratings of the items’ consistency with the Diagnostic and Statistical Manual of Mental Disorders (American Psychiatric Association 1994). We included the scales Negative affect (12 items); Anxiety (6 items); Oppositional Defiant Disorder (5 items); Attention Deficit Hyperactivity Disorder (5 items) and the 10-item CBCL-based autism scale as developed by So and colleagues (2013).

All DSM-oriented scales were scored by IRT (item response theory), using the R package ‘mirt’ (Chalmers 2012; R Core Team 2018). To this end we fitted a graded response model to the item responses of each scale (Samejima 1997), and saved the latent trait score for further analyses. The IRT model estimated the probability of an individual’s score on an item based on their latent trait score and the item's “difficulty level”. The latent trait scores were assumed to follow a normal distribution with a mean of 0 and a standard deviation of σ. The advantage of IRT to estimate latent trait scores is that it provides a more precise measurement of an individual's underlying trait by accounting for the difficulty level of the items and the individual's response pattern.

Intelligence scores were obtained by age-appropriate psychometric IQ tests: the Revised Amsterdam Child Intelligence Test (Bleichrodt et al. 1988; Bartels et al. 2002), WAIS (Weiss et al. 2010), or WISC (Sattler 1988; Wechsler et al. 2002). The participants’ ages differed over the different studies that collected IQ, ranging from 5 to 18 years. We selected IQ scores obtained when the twins were 7 years old, or as close as possible to age 7. The mean age at the time of IQ testing was 7.8 (median = 7, *SD* = 3.7). Within each wave of data collection, IQ scores were transformed to Z-scores, with a mean of 0 and a variance and SD of 1.

### Statistical Analyses

First, we obtained the descriptive statistics (means and variances) and the correlation matrix of intelligence and the five psychopathology scales. Since the presence of a nonlinear relation between moderator and outcome can give rise to spurious moderation results (Rathouz et al. 2008), we tested for both linear and non-linear associations of intelligence (IQ) by fitting regression models in which the psychopathology scales are predicted by z-scored IQ (zIQ) and z-scored IQ squared (zIQ^2^). We applied general estimation equations (GEE) to correct the standard errors of the regression coefficients for the dependence in the data (i.e., twins nested in pairs).

Next, we estimated the environmental and genetic contributions to the phenotypic variance of the DSM-oriented psychopathology scales and intelligence in bivariate models, which we fitted using raw data maximum likelihood estimation. The variance decomposition models were separately fitted to the largest datasets, i.e., including all 14,150 twin pairs with CBCL data and 621 with IQ data (including all incomplete pairs). We included additive (A) genetic influences and person-unique environment (E) in all models and included either shared environment (C) or genetic non-additive factors (D), depending on the pattern of MZ and DZ correlations (Posthuma et al. 2003), and tested for the significance of C or D by loglikelihood ratio tests. The *Supplementary Methods* offers more detail about the uni- and bivariate twin models, and the bivariate model with moderation.

We tested in bivariate twin models whether the A, C/D, and E influences were shared between intelligence and the psychopathology variables, and whether A, C/D, and E influences on psychopathology were moderated by intelligence (Purcell 2002; van der Sluis et al. 2012). We tested whether the psychopathology scores were affected by sex, and whether the IQ scores were affected by the age at which the IQ data were collected. If so, we used the confounding variable(s) as a covariate in further analyses.

### Addressing Nonnormality

**Fig. 2:**
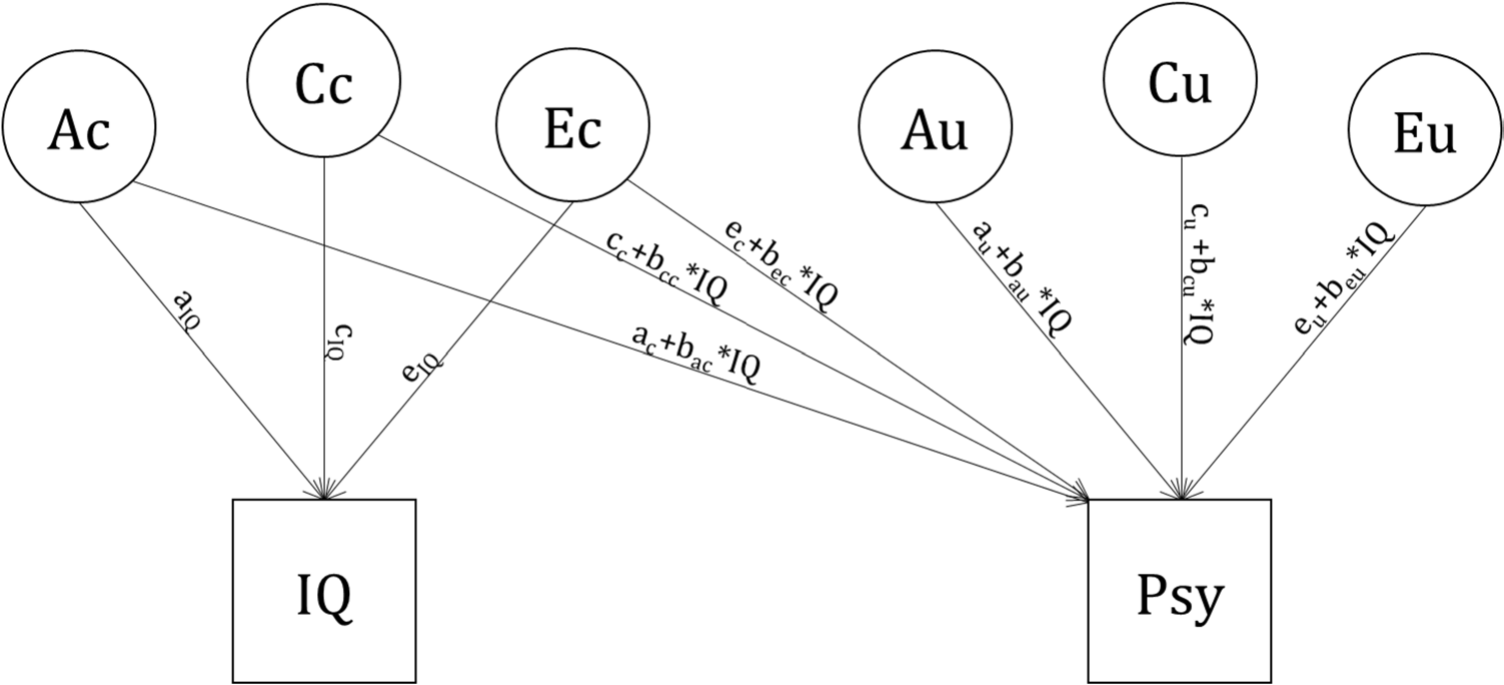
Bivariate moderation twin model. The moderator is measured IQ, and the phenotype of interest is a psychopathology phenotype (Psy). Fixed effects of sex and age are not shown. A, C and E are latent additive genetic, Common and Unique environmental influences, subscripts c and u denote influences common to the 2 phenotypes and unique to psychopathology, respectively

Moderation models involve a test of interaction. For instance, if IQ influences the genetic effects on psychopathology, i.e., b_ac_ ≠ 0 and/or b_au_ ≠ 0 in Fig. 2, this can be interpreted as a A-by-IQ interaction, where the heritability of the psychopathology phenotype depends on IQ. Estimated moderation effects may depend on the scale of the variables. Specifically, non-normality can give rise to spurious moderation/interaction effects (Purcell 2002; Eaves 2006; Eaves and Verhulst 2014). In the present case, we recognize that, even while working with IRT scores, the psychopathology variables display floor effects, i.e., appear to be left censored (see Supplementary Figures). To take this into account, we modeled these variables explicitly as left censored by basing the likelihood function on the censored bivariate normal distribution. In this distribution, we modeled a fixed censoring threshold based on the proportion of minimum-scores (see also de Zeeuw et al. 2019; Kevenaar et al. 2023).

### Deviations from the Pre-Registered Analyses

In our pre-registration, we described two steps to address nonnormality: trichotomizing and re-analyzing the data via a liability-threshold model, and Box-Cox transforming the data. Trichotomizing leads to a decrease in statistical power (1-β), i.e., a high probability β of false negative results, which was undesirable given the sample size for the bivariate analyses. Second, we found that the Box-Cox transformations did not normalize the distributions. Therefore, we did not pursue these options and replaced these steps with the censoring correction approach described above.

We pre-registered a significance threshold based on the number of tests, corrected for the covariance between variables (Li and Ji 2005). This led to an alpha of 0.001, which is overly strict, given the ultimate number of complete twin pairs with both CBCL and IQ data this would result in a severely underpowered test. Therefore, we corrected for the number of tests performed for each psychopathology phenotype within each statistical model. This led to a better balance between the false positive rate (α) and the false negative rate (β). We had also preregistered a longitudinal analysis that turned out to be unfeasible due to the limited number of twin pairs with complete longitudinal CBCL and IQ data. Therefore, we did not perform this analysis.

We added an additional test that we had not pre-registered, i.e., we reversed the moderation model to explore whether genetic and environmental effects on intelligence are moderated by psychopathology. This was done to explore reciprocal moderation effects of IQ on psychopathology and of psychopathology on IQ.

## Results

**Fig. 3:**
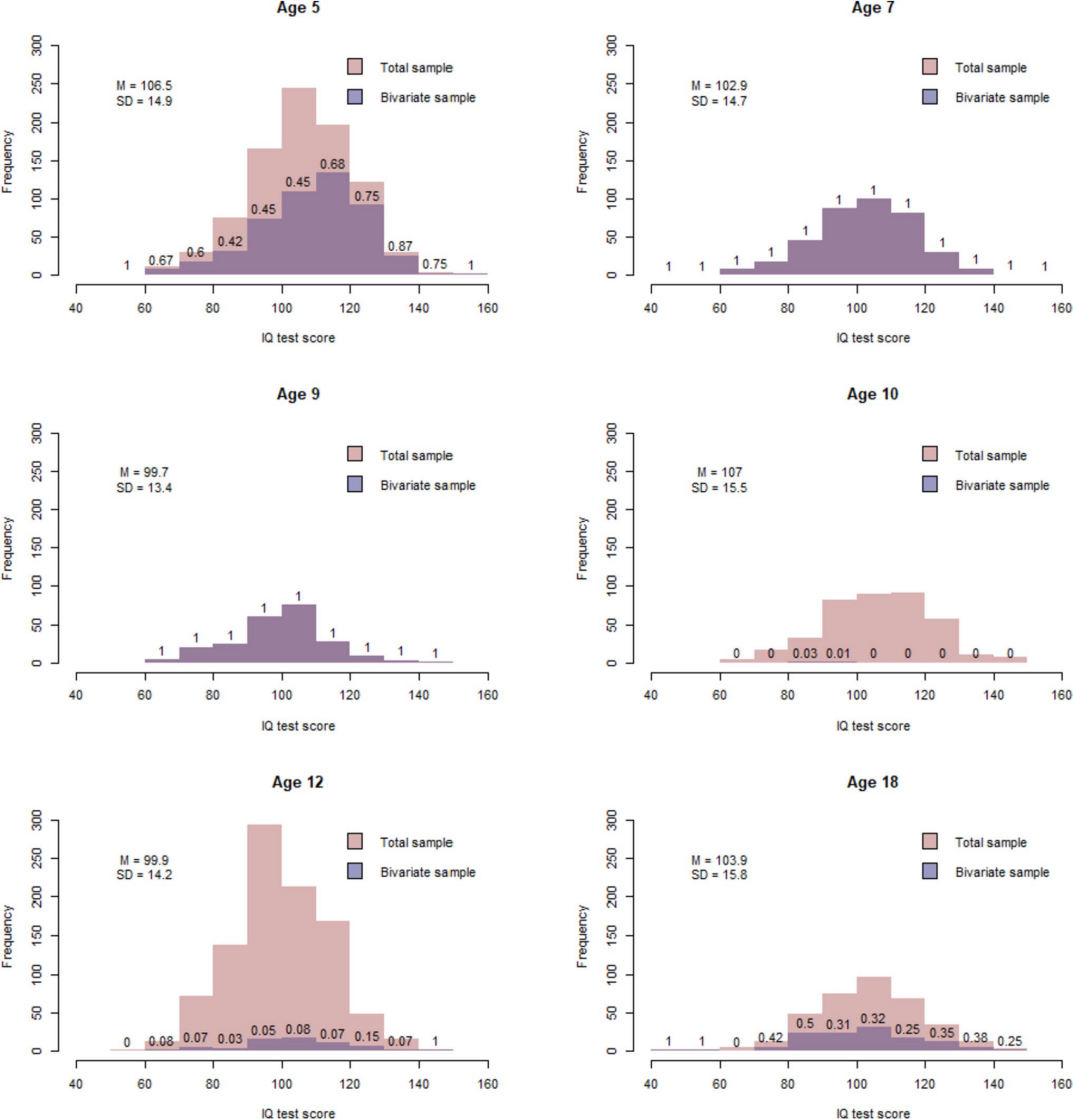
Histograms of IQ scores, by age. The x-axes display IQ score in bins of 10 points from 40 to 160, the y-axes display their frequency. Numbers on the bars indicate the proportions of participants included in the analyses. The total sample refers to all participants with IQ data at a particular age. The bivariate sample refers to children whose data were included in the bivariate analyses of IQ and psychopathology. We selected IQ data obtained at age 7 years, or as close as possible to age 7

Descriptive statistics for the psychopathology variables are presented in Table 1. Figure 3 displays the distributions, means, standard deviations of IQ, and the proportion of IQ data at each age include in the sample for the bivariate analyses. Cross-twin-cross-trait correlations are presented in Supplementary Table 1 & 2.

**Table 1:** Descriptive statistics by psychopathology phenotype for the total group and by sex.

Phenotype	Total group			Boys			Girls		
	*M*	*SD*	*n*	*M*	*SD*	*n*	*M*	*SD*	*n*
Negative affect	0.70	1.44	28,236	0.70	1.45	14,211	0.70	1.44	14,025
Anxiety	0.67	1.31	28,235	0.65	1.31	14,210	0.68	1.30	14,025
ODD	1.23	1.84	28,235	1.36	1.97	14,210	1.10	1.69	14,025
ADHD	1.10	1.85	28,236	1.26	2.02	14,211	0.93	1.65	14,025
Autism	0.75	1.51	28,239	0.85	1.66	14,212	0.64	1.33	14,027

Means and SDs are based on sum scores

All five psychopathology variables correlated negatively with IQ, with correlations ranged from − 0.09 to − 0.15. There was no evidence for nonlinearity in the correlations between IQ and psychopathology (see Supplementary Table 3 and 4). All psychopathology phenotypes were influenced by additive genetic factors and by an unshared environment. Negative affect and ODD were influenced by shared environment. For autism and ADHD, we found significant contributions of genetic dominance effects. The standardized variance components are presented in Table 2.

**Table 2:** Standardized variance components for influence of A, C/D and E, and 95% confidence intervals (based on the best fitting univariate models).

Phenotype	σ_A_^2^	σ_C_^2^	σ_D_^2^	σ_E_^2^
Negative affect	.54 (.50, −.58)	.12 (.09, −.16)	–	.34 (.33, −.35)
Anxiety	.60 (.55, −.64)	.00 (−.02, −.05)	–	.39 (.38, −.41)
ODD	.60 (.56, −.63)	.16 (.11, −.19)	–	.25 (.24, −.26)
ADHD	.18 (.10, −.26)	–	.53 (.46, −.62)	.28 (.27, −.29)
Autism	.28 (.20, −.36)	–	.33 (.25, −.41)	.39 (.37, −.40)
IQ	.37 (.20, −.55)	.33 (.17, −.48)	–	.30 (.25, −.35)

Standardized variance components are rounded to the nearest two decimals and may not add up to exactly 1. See Supplementary Table 5 for model fitting results

### Bivariate Analyses of Psychopathology and IQ

**Fig. 4:**
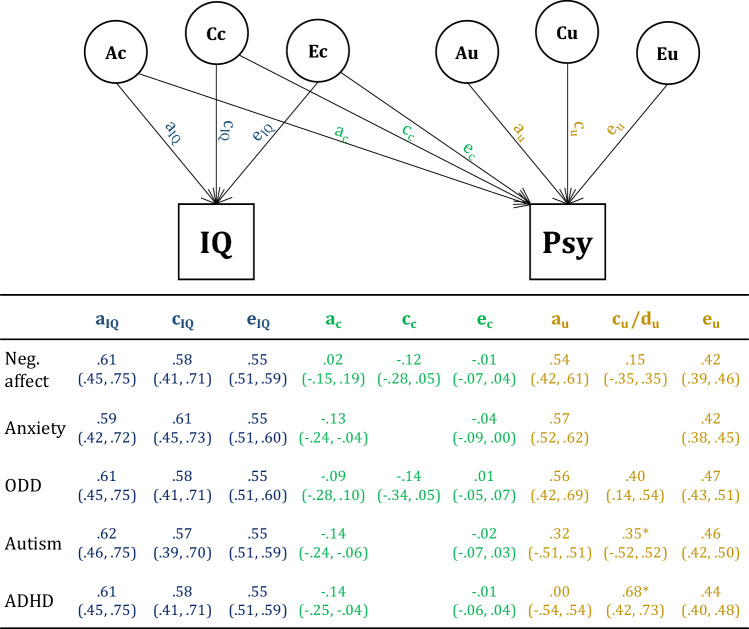
Raw parameter estimates of bivariate analyses of IQ and the five psychopathology phenotypes, and 95% confidence intervals (in brackets). *Autism and ADHD are influenced by genetic dominance (d_u_).

Covariance between anxiety, ADHD, and autism on the one hand, and IQ on the other hand, were due only to common genetic factors, where additive genetic influences on IQ were negatively related to psychopathology (χ^2^ (Δdf) = 7.84 (1), *p* = 0.005 for anxiety, χ^2^ (Δdf) = 8.11 (1), *p* = 0.004 for ADHD, and χ^2^ (Δdf) = 12.64 (1), *p* =  < 0.001 for autism). For negative affect and ODD, none of the individual cross paths were significantly different from zero, implying that we did not detect any common genetic or environmental factors driving the covariance between anxiety and ODD, and IQ. To reduce model complexity and improve statistical power, nonsignificant cross paths were not included in subsequent models. Parameter estimates are presented in Fig. 4. Phenotypic correlations between IQ and psychopathology—as estimated in the bivariate twin model—are presented in Table 3.

**Table 3:** A, C, and E correlations and phenotypic correlations (P) between IQ and psychopathology, as estimated in bivariate analyses of IQ and psychopathology, and 95% confidence intervals.

Phenotype	A	C	E	P
Negative affect	.01 (− .12, .14)	− .10 (− .19, .04)	− .01 (− .07, .02)	− .09 (− .37, .20)
Anxiety	**− .11 (**−**.18, −.03)**		− .03 (− .09, .00)	− .14 (− .26, -.03)
ODD	− .06 (− .22, .07)	− .09 (− .23, .03)	.01 (− .05, .03)	− .15 (− .48, .12)
ADHD	− **.14 ** **(**−**.16, −.04)**		− .01 (− .06, .02)	− .14 (− .21, .03)
Autism	− **.11 ** **(**−**.17, −.02)**		− .01 (− .05, .02)	− .11 (− .22, -.01)

**Boldfaced** estimates are significant. Correlations and 95% confidence intervals are rounded to the nearest 2 integers. See Supplementary Table 6 for model fitting results

### Moderation Analyses of Psychopathology and IQ

**Fig. 5:**
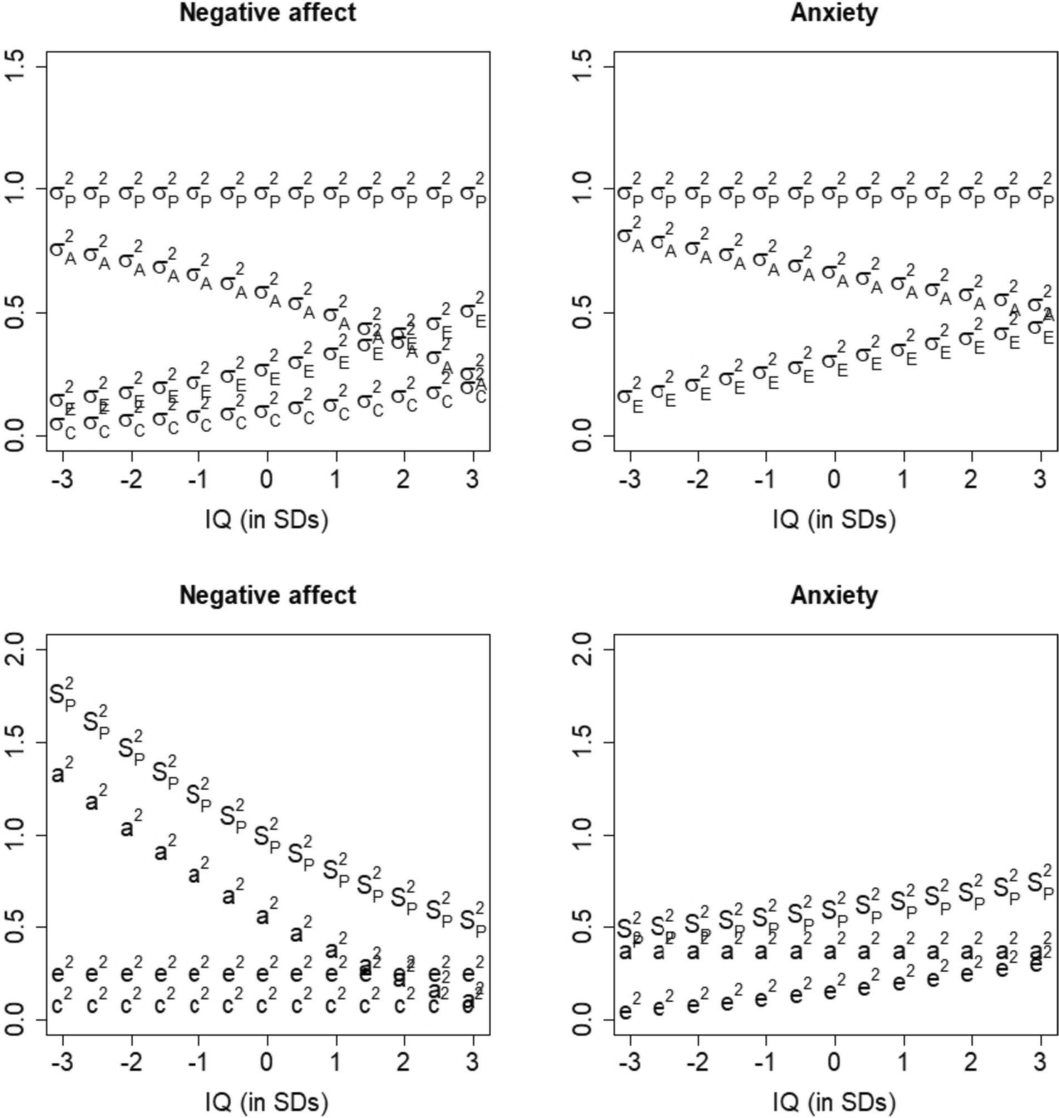
Means and variance components for negative affect (left) and anxiety (right), conditional on IQ. The upper two figures display standardized variance components, the lower two figures display unstandardized variance components. These estimates are based on the analyses with censoring correction. S_P_^2^ and σ_P_^2^ are total variance; a^2^ and σ_A_^2^ are additive genetic variance; c^2^ and σ_C_^2^ are environmental variance shared between members of a twin pair; and e^2^ and σ_E_.^2^ are environmental variance not shared between members of a twin pair. Note that IQ is standardized according to sample mean and SD, and as such, the standardized IQ scores reflect the sample mean and SD, rather than a mean of 100 and a SD of 15

**Fig. 6:**
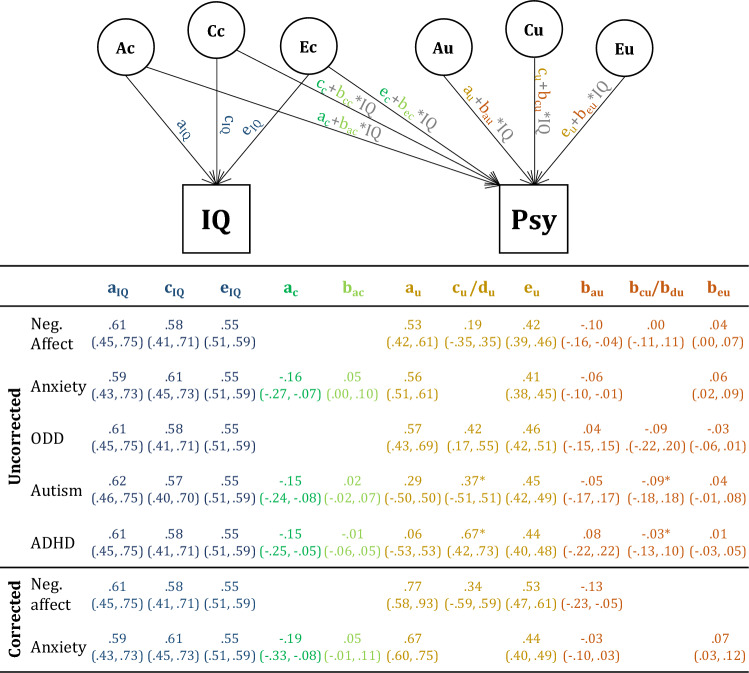
Raw parameter estimates from moderation analyses of IQ and psychopathology and 95% confidence intervals (in brackets). ‘Uncorrected’ and ‘corrected’ refer to the analyses uncorrected and corrected for censoring. *Autism and ADHD are influenced by genetic dominance (d_u_ and b_du_)

IQ moderated the additive genetic effects on negative affect and anxiety and the effects of the unique environment on anxiety. To examine the influence of violation of distributional assumptions, we re-analyzed the data on anxiety and negative affect while correcting for censoring. Only the moderation test of the additive genetic effect on negative affect (χ^2^ (Δdf) = 9.30 (1), *p* = 0.002), and the unique environmental effect on anxiety remained significant (χ^2^ (Δdf) = 9.14 (1), *p* = 0.003). The complete model fitting results are presented in Supplementary Tables 7 and 8. The moderation entails that the additive genetic effects on negative affect declined with increasing IQ, and unique environmental effects on anxiety increase with increasing IQ. Standardized and unstandardized variance components for negative affect and anxiety as a function of IQ are shown in Fig. 5. The standardized and unstandardized variance components convey different sources of information: Standardized variance components reflect the proportion of total phenotypic variance due to A, C, and E, from which we see how the relative contributions of genetic and environmental effects change with increasing intelligence. Unstandardized variance components reflect the raw, absolute variance due to A, C, and E, from which we see what the source of this change is. In the case of negative affect and anxiety, the bottom of Fig. 5 shows that the *relative* genetic variance (σ_A_^2^) of both negative affect and anxiety decreases with increasing IQ, and the relative environmental variance (σ_C_^2^, σ_E_^2^) increases with increasing IQ. In the top of Fig. 5, we see that this is due to different sources: The raw genetic variance (a^2^) of negative affect decreases with increasing IQ, but the raw environmental variance (c^2^, e^2^) does not change; while the raw genetic variance of anxiety does not change with increasing IQ, but the raw environmental variance increases. Parameter estimates are presented in Fig. 6.

To test for reciprocal moderation effects, we reversed the moderation model. The results of these analyses, in which psychopathology is allowed to moderate the genetic and environmental effects on IQ, are shown in Supplementary Table 9. We found no evidence for such moderation.

## Discussion

We confirmed the negative association between intelligence and five indices of psychopathology. We first estimated this association in a population-based sample of seven-year-old twins, and next tested if this relation was attributable to shared genetic and environmental influences. Finally, we tested whether genetic and environmental influences on psychopathology were moderated by intelligence, and whether genetic and environmental effects on intelligence were moderated by psychopathology. Such moderation, if detected, would imply that the contribution of genetic and environmental factors to individual differences in psychopathology differs as a function of intelligence, or that the contribution of genetic and environmental factors to individual differences in intelligence differs as a function of psychopathology.

We found that intelligence correlated negatively with negative affect, anxiety, ODD, ADHD, and Autism. These correlations in part reflected common genetic effects, with genetic factors that increase intelligence decrease psychopathology. Genetic and environmental effects on negative affect and anxiety (respectively) were moderated by intelligence, such that the heritability of both anxiety and negative affect was greatest in children with lower IQ. For negative affect, this is because the genetic variance increases with decreasing intelligence, while the environmental variance is the same over the range of intelligence. For anxiety, this is because the environmental variance increases with increasing intelligence, while the additive genetic variance is the same over the range of intelligence. We found no evidence that genetic and environmental effect on intelligence were moderated by psychopathology.

Genetic factors that decrease intelligence, increase symptoms of anxiety, ADHD, and autism, suggesting that the relations between intelligence on the one hand, and anxiety, ADHD, and autism on the other hand, are either driven by a common downstream mechanism or a causal relation, with intelligence causally affecting psychopathology, or vice versa.

The finding that intelligence moderates genetic influences on negative affect and environmental influences on anxiety suggests that 7 yead old children with higher intelligence are less sensitive to genetic factors that predispose them to affective psychopathology than children with lower intelligence. On the other hand, children with lower intelligence are more sensitive to environmental factors that contribute to childhood anxiety.

While we cannot identify specific risk factors based on these results, a candidate endophenotype underlying the common genetic factors is executive functioning, which has been linked to both intelligence and various psychopathologies (Kusche et al. 1993; Pennington and Ozonoff 1996; Stins et al. 2005; Polderman et al. 2006; Martel et al. 2007; Harden et al. 2020). Executive functions are cognitive functions involved in context-specific action selection, such as response inhibition, planning, and working memory (Pennington and Ozonoff 1996). For example, Kusche et al. (1993) reported deficits in executive function for children with internalizing (anxiety and somatic problems), and externalizing (ADHD, and conduct problems) problems. Harden et al. (2020) reported consistent genetic correlations between executive functions, intelligence, and general psychopathology, indicating pleiotropic effects.

The moderation results imply that individuals with (above) average intelligence are better protected against risk factors for negative affect and anxiety, or that individuals with below-average intelligence experience different environmental circumstances than individuals with (above-) average intelligence (Hassiotis et al. 2019; Smith et al. 2021). There is some evidence that genetic effects on anxiety and affective psychopathology are amplified by adverse experiences (Wang et al. 2023).

This study has the following limitations. Although the dataset on psychopathology was large, the bivariate sample size was relatively small (in terms of complete pairs: 285 MZ twin pairs and 331 DZ pairs). Consequently, we may have been unable to detect small effects. While the correlations between IQ and all five indicators of psychopathology are negative, we found no statistically significant contribution of genetic or environmental factors to the relationship between IQ, on the one hand, and negative affect and ODD, on the other hand. We attribute this to the number of parameters estimated in the bivariate twin design in combination with our sample size: The tests were likely underpowered to detect (genetic and environmental contributions to) a correlation between psychopathology and intelligence, whereas we did detect this association in straightforward linear regression analysis (using general estimation equations). Another limitation due to sample size constraints is that we did not study developmental processes, i.e., we did not analyze longitudinal data.

We recognize that moderation is a scale dependent statistical phenomenon, and any detected moderation/interaction effects might be due to scaling, rather than true effects. Scaling can be a source of concern in two ways: First, violation of distributional assumptions can be a source of false positive moderation results (Purcell 2002; Eaves 2006; Verhulst et al. 2019). Second, interaction effects are said to be scale-dependent when these effects appear or disappear after a monotonic transformation of the outcome data. Loftus (1978) and Wagenmakers et al. (2012) note that interactions that are not scale dependent remain after a monotonic non-linear transformation of the measurement scale. Scale-dependent interactions can thus be removed by such a transformation, which can be employed as a test of scale-dependency. However, the floor effect in the CBCL data, reflecting children who do not have any psychopathology symptoms, cannot be transformed away. Therefore, we applied a censored distribution model. Based on these results, we are confident that our findings are not the result of violation of the normality assumption, but we still cannot rule out that our results are due to scale-dependency.

Rathouz et al. (2008) also discuss several ways in which the bivariate moderation model can produce spurious moderation results. This can occur when the moderator also moderates the covariance between moderator and phenotype (in the bivariate moderation model: moderation of the ac, cc, and ec paths), or when the relation between phenotype and moderator is nonlinear. In our data, there were no nonlinear relations between IQ and any of the psychopathologies, and the covariance paths (ac, cc, ec) were not moderated by IQ. Therefore, these sources of spurious results are unlikely apply to our present results.

To our knowledge, we are the first to study whether genetic and environmental influences on psychopathology depend on intelligence. In a different study, we aimed to replicate these results by testing whether environmental effects on anxiety and negative affect were moderated by a polygenic score of intelligence (Environment-by-PGS interaction; Bruins et al. 2023). Results from this study indicate that environmental effects on negative affect were moderated by genetic effects on intelligence, but there was no evidence that environmental effects on anxiety were moderated. Replication of these results is warranted with future research also focusing on identifying protective and risk factors for psychopathology that are particularly relevant for individuals with below-average intellectual functioning. Here, we highlight adverse life events, coping skills and executive function deficits as potential risk (endo)phenotypes. Insight in how these and other factors operate in the development of psychopathology across the range of intelligence could inform prevention and treatment strategies.

### Supplementary Information

Below is the link to the electronic supplementary material.

Supplementary file1 (DOCX 58 kb)

Supplementary file2 (DOCX 42 kb)

Supplementary file3 (PDF 1145 kb)

## Data Availability

Participants did not agree for their data to be shared publicly. The data are available on request, for reproducibility purposes.

## Code Availability

Scripts for the analyses are available on OpenScience Framework (OSF) via 10.17605/OSF.IO/4DEGN
