# Supplementary material for: Are Genetic and Environmental Risk Factors for Psychopathology Amplified in Children with Below-Average Intelligence? A Population-Based Twin Study
Source: Behav Genet. 2024 Feb 14;54(3):278–89. doi: 10.1007/s10519-023-10174-7 (PMC11032279; doi:10.1007/s10519-023-10174-7)
Supplement: Supplementary file 1 — Supplementary file1 (DOCX 58 kb) [file 10519_2023_10174_MOESM1_ESM.docx]

**Supplementary Tables.**

Are genetic and environmental risk factors for psychopathology amplified in children with below-average intelligence? A population-based twin study.

Behavior Genetics

Susanne Bruins^1,2^, Elsje van Bergen^1,2,3^, Maurits W. Masselink^4,5,6^, Stefania A. Barzeva^4,6^

Catharina A. Hartman^6^, Roy Otten^4^, Nanda N.J. Rommelse^6,7^,

Conor V. Dolan^1^ & Dorret I. Boomsma^1,8^

^1^ Department of Biological Psychology, Vrije Universiteit Amsterdam, the Netherlands
^2^ Amsterdam Public Health Research Institute, Amsterdam, The Netherlands
^3^ Research Institute LEARN!, Vrije Universiteit Amsterdam
^4^ Radboud University, Behavioural Science Institute, Nijmegen, The Netherlands
^5^ Radboud University Medical Center, Department of Psychiatry, Nijmegen, The Netherlands
^6^ Department of Psychiatry, University of Groningen, University Medical Center Groningen, Groningen, The Netherlands
^7^ Karakter Child and Adolescent Psychiatry University Center, Nijmegen, The Netherlands
^8^Amsterdam Research and Development (AR&D) Research Institute, Amsterdam, The Netherlands

Corresponding Author: Susanne Bruins, s.bruins@vu.nl

*Supplementary Table 1.*

MZ twin correlations within and between psychopathology at age 7 and IQ.

|  |  | **Aff.** | | **Anx.** | | **ADHD** | | **Autism** | | **ODD** | | **IQ** | |
| --- | --- | --- | --- | --- | --- | --- | --- | --- | --- | --- | --- | --- | --- |
|  |  | **T1** | **T2** | **T1** | **T2** | **T1** | **T2** | **T1** | **T2** | **T1** | **T2** | **T1** | **T2** |
| **Aff.** | **T1** | 1 | 0,62 | 0,52 | 0,37 | 0,36 | 0,33 | 0,36 | 0,33 | 0,45 | 0,39 | -0,08 | -0,11 |
|  | **T2** | 0,62 | 1 | 0,44 | 0,55 | 0,37 | 0,35 | 0,27 | 0,40 | 0,42 | 0,40 | -0,14 | -0,19 |
| **Anx.** | **T1** | 0,52 | 0,44 | 1 | 0,66 | 0,34 | 0,34 | 0,29 | 0,31 | 0,31 | 0,28 | -0,17 | -0,14 |
|  | **T2** | 0,37 | 0,55 | 0,66 | 1 | 0,32 | 0,40 | 0,20 | 0,41 | 0,31 | 0,33 | -0,12 | -0,14 |
| **ADHD** | **T1** | 0,36 | 0,37 | 0,34 | 0,32 | 1 | 0,72 | 0,31 | 0,34 | 0,54 | 0,50 | -0,08 | -0,13 |
|  | **T2** | 0,33 | 0,35 | 0,34 | 0,40 | 0,72 | 1 | 0,36 | 0,42 | 0,42 | 0,56 | -0,08 | -0,15 |
| **ODD** | **T1** | 0,36 | 0,27 | 0,29 | 0,20 | 0,31 | 0,36 | 1 | 0,54 | 0,28 | 0,27 | -0,15 | -0,19 |
|  | **T2** | 0,33 | 0,40 | 0,31 | 0,41 | 0,34 | 0,42 | 0,54 | 1 | 0,32 | 0,28 | -0,08 | -0,15 |
| **Autism** | **T1** | 0,45 | 0,42 | 0,31 | 0,31 | 0,54 | 0,42 | 0,28 | 0,32 | 1 | 0,68 | -0,14 | -0,23 |
|  | **T2** | 0,39 | 0,40 | 0,28 | 0,33 | 0,50 | 0,56 | 0,27 | 0,28 | 0,68 | 1 | -0,13 | -0,22 |
| **IQ** | **T1** | -0,08 | -0,14 | -0,17 | -0,12 | -0,08 | -0,08 | -0,15 | -0,08 | -0,14 | -0,13 | 1 | 0,72 |
|  | **T2** | -0,11 | -0,19 | -0,14 | -0,14 | -0,13 | -0,15 | -0,19 | -0,15 | -0,23 | -0,22 | 0,72 | 1 |

note. *Aff* stands for negative affect, *anx* stands for anxiety. Correlations are rounded to the nearest two decimals.

*Supplementary Table 2.*

DZ twin correlations within and between psychopathology at age 7 and IQ.

|  |  | **Aff.** | | **Anx.** | | **ADHD** | | **Autism** | | **ODD** | | **IQ** | |
| --- | --- | --- | --- | --- | --- | --- | --- | --- | --- | --- | --- | --- | --- |
|  |  | **T1** | **T2** | **T1** | **T2** | **T1** | **T2** | **T1** | **T2** | **T1** | **T2** | **T1** | **T2** |
| **Aff.** | **T1** | 1 | 0,38 | 0,47 | 0,3 | 0,29 | 0,29 | 0,45 | 0,19 | 0,42 | 0,28 | -0,06 | -0,01 |
|  | **T2** | 0,38 | 1 | 0,21 | 0,42 | 0,17 | 0,33 | 0,14 | 0,42 | 0,23 | 0,35 | -0,12 | -0,05 |
| **Anx.** | **T1** | 0,47 | 0,21 | 1 | 0,33 | 0,25 | 0,19 | 0,34 | 0,14 | 0,37 | 0,27 | -0,04 | 0,09 |
|  | **T2** | 0,30 | 0,42 | 0,33 | 1 | 0,12 | 0,29 | 0,23 | 0,54 | 0,28 | 0,29 | -0,03 | -0,16 |
| **ADHD** | **T1** | 0,29 | 0,17 | 0,25 | 0,12 | 1 | 0,18 | 0,22 | 0,22 | 0,56 | 0,32 | -0,09 | 0 |
|  | **T2** | 0,29 | 0,33 | 0,19 | 0,29 | 0,18 | 1 | 0,26 | 0,39 | 0,33 | 0,62 | -0,07 | -0,11 |
| **ODD** | **T1** | 0,45 | 0,14 | 0,34 | 0,23 | 0,22 | 0,26 | 1 | 0,20 | 0,26 | 0,24 | -0,15 | -0,08 |
|  | **T2** | 0,19 | 0,42 | 0,14 | 0,54 | 0,22 | 0,39 | 0,20 | 1 | 0,38 | 0,38 | -0,12 | -0,16 |
| **Autism** | **T1** | 0,42 | 0,23 | 0,37 | 0,28 | 0,56 | 0,33 | 0,26 | 0,38 | 1 | 0,49 | -0,14 | -0,07 |
|  | **T2** | 0,28 | 0,35 | 0,27 | 0,29 | 0,32 | 0,62 | 0,24 | 0,38 | 0,49 | 1 | -0,17 | -0,13 |
| **IQ** | **T1** | -0,06 | -0,12 | -0,04 | -0,03 | -0,09 | -0,07 | -0,15 | -0,12 | -0,14 | -0,17 | 1 | 0,50 |
|  | **T2** | -0,01 | -0,05 | 0,09 | -0,16 | 0 | -0,11 | -0,08 | -0,16 | -0,07 | -0,13 | 0,50 | 1 |

note. *Aff* stands for negative affect, *anx* stands for anxiety. Correlations are rounded to the nearest two decimals.

*Supplementary Table 3.*

Pearson’s correlations between IQ and psychopathology.

| **Negative affect** | | **Anxiety** | **ADHD** | **Autism** | **ODD** | **IQ** |
| --- | --- | --- | --- | --- | --- | --- |
| **Negative affect** | 1.00 |  |  |  |  |  |
| **Anxiety** | 0.48 | 1.00 |  |  |  |  |
| **ADHD** | 0.33 | 0.32 | 1.00 |  |  |  |
| **Autism** | 0.41 | 0.40 | 0.33 | 1.00 |  |  |
| **ODD** | 0.40 | 0.33 | 0.57 | 0.30 | 1.00 |  |
| **IQ** | -0.09 | -0.12 | -0.11 | -0.15 | -0.15 | 1.00 |

*Supplementary Table 4*.

Linear and nonlinear relations between IQ and psychopathology

|  | **predictor** | ***Intercept*** | ***b*** | ***Z*** | ***S.E.*** | ***p*** |
| --- | --- | --- | --- | --- | --- | --- |
| **Negative affect** | IQ | 0.020 | -0.050 | -2.281 | 0.022 | .011 |
|  | IQ^2^ |  | -0.002 | -0.180 | 0.013 | .429 |
| **Anxiety** | IQ | -0.031 | -0.098 | -4.220 | 0.023 | <.001 |
|  | IQ^2^ |  | 0.024 | 1.624 | 0.014 | .948 |
| **ODD** | IQ | 0.027 | -0.094 | -3.540 | 0.027 | <.001 |
|  | IQ^2^ |  | -0.002 | -0.130 | 0.015 | .448 |
| **Autism** | IQ | -0.091 | -0.090 | -4.194 | 0.022 | <.001 |
|  | IQ^2^ |  | -0.017 | 1.348 | 0.013 | .911 |
| **ADHD** | IQ | 0.077 | -0.082 | -3.146 | 0.026 | <.001 |
|  | IQ^2^ |  | 0.007 | 0.445 | 0.015 | .672 |

note. Parameters were estimated with general estimation equations (GEE). Z is the robust Z-statistic, S.E. is the robust standard error.

Supplementary Table 5.

Results of univariate twin model fitting.

| **Phenotype** | **Model** | **-2LL** | **df** | **χ^2^** | **Δdf** | ***p*** | **AIC** |
| --- | --- | --- | --- | --- | --- | --- | --- |
| **Negative affect** | **ACE** | 55965.09 | 28231 |  |  |  | -496.91 |
|  | **AE** | 56010.37 | 28232 | 45.29 | 1 | <.001 | -453.63 |
| **Anxiety** | **ACE** | 60438.97 | 28230 |  |  |  | 3978.97 |
|  | **AE** | 60439.49 | 28231 | 0.52 | 1 | .470 | 3977.49 |
| **ODD** | **ACE** | 64991.17 | 28230 |  |  |  | 8531.17 |
|  | **AE** | 65071.30 | 28231 | 80.13 | 1 | <.001 | 8609.30 |
| **Autism** | **ADE** | 59777.96 | 28234 |  |  |  | 3309.96 |
|  | **AE** | 59845.92 | 28235 | 67.96 | 1 | <.001 | 3375.92 |
| **ADHD** | **ADE** | 64916.90 | 28231 |  |  |  | 8454.90 |
|  | **AE** | 65104.13 | 28232 | 187.22 | 1 | <.001 | 8640.13 |
| **IQ** | **ACE** | 3225.73 | 1233 |  |  |  | 759.73 |
|  | **AE** | 3239.34 | 1234 | 13.62 | 1 | <.001 | 771.34 |

α = .025. -2LL is the -2 loglikelihood, χ^2^ is the -2LL ratio test.

Supplementary Table 6.

Results of bivariate twin model fitting.

| **Phenotype** | **Test** | **-2LL** | **df** | **χ^2^** | **Δdf** | ***p*** | **AIC** |
| --- | --- | --- | --- | --- | --- | --- | --- |
| **Negative affect^1^** |  | 5381.81 | 2313 |  |  |  | 755.81 |
|  | **a_c_** | 5381.86 | 2314 | 0.05 | 1 | .824 | 753.86 |
|  | **c_c_** | 5383.80 | 2314 | 1.99 | 1 | .158 | 755.80 |
|  | **e_c_** | 5382.15 | 2314 | 0.34 | 1 | .562 | 754.15 |
| **Anxiety^2^** |  | 5395.17 | 2315 |  |  |  | 765.17 |
|  | **a_c_** | 5403.01 | 2316 | 7.84 | 1 | .005 | 771.01 |
|  | **e_c_** | 5398.56 | 2316 | 3.39 | 1 | .066 | 766.56 |
| **ODD^1^** |  | 5702.47 | 2313 |  |  |  | 1076.47 |
|  | **a_c_** | 5703.33 | 2314 | 0.86 | 1 | .355 | 1075.33 |
|  | **c_c_** | 5704.69 | 2314 | 2.22 | 1 | .136 | 1076.69 |
|  | **e_c_** | 5702.59 | 2314 | 0.12 | 1 | .724 | 1074.59 |
| **ADHD^2^** |  | 5676.68 | 2314 |  |  |  | 1048.68 |
|  | **a_c_** | 5684.79 | 2315 | 8.11 | 1 | .004 | 1054.79 |
|  | **e_c_** | 5676.72 | 2315 | 0.04 | 1 | .833 | 1046.72 |
| **Autism^2^** |  | 5340.94 | 2315 |  |  |  | 710.94 |
|  | **a_c_** | 5353.58 | 2316 | 12.64 | 1 | <.001 | 721.58 |
|  | **e_c_** | 5341.35 | 2316 | 0.41 | 1 | .523 | 709.35 |

^1^α = .017, ^2^α = .025. -2LL is the -2 loglikelihood, χ^2^ is the -2LL ratio test.

*Supplementary Table 7.*

Results of bivariate twin moderation model fitting, uncorrected for censoring

| **Phenotype** | **Test** | **-2LL** | **df** | **Δχ^2^** | **Δdf** | ***p*** | **AIC** |
| --- | --- | --- | --- | --- | --- | --- | --- |
| **Negative Affect^1^** |  | 5372.65 | 2313 |  |  |  | 746.65 |
|  | **b_au_** | 5382.36 | 2314 | 9.71 | 1 | 0.002 | 754.36 |
|  | **b_cu_** | 5372.65 | 2314 | <0.01 | 1 | 0.961 | 744.65 |
|  | **b_eu_** | 5376.30 | 2314 | 3.65 | 1 | 0.056 | 748.30 |
| **Anxiety^2^** |  | 5383.58 | 2313 |  |  |  | 757.58 |
|  | **b_au_+b_ac_** | 5395.63 | 2315 | 12.05 | 2 | 0.002 | 765.63 |
|  | **b_eu_** | 5393.25 | 2314 | 9.68 | 1 | 0.002 | 765.25 |
| **ODD^1^** |  | 5716.64 | 2313 |  |  |  | 1090.64 |
|  | **b_au_** | 5716.82 | 2314 | 0.18 | 1 | 0.671 | 1088.82 |
|  | **b_cu_** | 5716.96 | 2314 | 0.32 | 1 | 0.570 | 1088.96 |
|  | **b_eu_** | 5718.47 | 2314 | 1.83 | 1 | 0.177 | 1090.47 |
| **Autism^1^** |  | 5321.30 | 2312 |  |  |  | 697.30 |
|  | **b_au_+b_ac_** | 5322.73 | 2314 | 1.43 | 2 | 0.489 | 694.73 |
|  | **b_du_** | 5322.54 | 2313 | 1.24 | 1 | 0.265 | 696.54 |
|  | **b_eu_** | 5324.04 | 2313 | 2.74 | 1 | 0.098 | 698.04 |
| **ADHD^1^** |  | 5675.87 | 2311 |  |  |  | 1053.87 |
|  | **b_au_+b_ac_** | 5676.04 | 2313 | 0.17 | 2 | 0.919 | 1050.04 |
|  | **b_du_** | 5676.00 | 2312 | 0.13 | 1 | 0.714 | 1052.00 |
|  | **b_eu_** | 5676.07 | 2312 | 0.21 | 1 | 0.650 | 1052.07 |

^1^α = .017, ^2^α = .025. Subscript au, cu, du, and eu stand for additive genetic, shared environmental, dominance genetic, and unique environmental effects unique to the psychopathology variable, and ac, cc, dc, and ec stand for additive genetic, shared environmental, dominance, and unique environmental effects that are common to both intelligence and psychopathology.

Supplementary Table 8.

Results of bivariate twin moderation model fitting, after correcting for censoring.

| **Phenotype** | **Test** | **-2LL** | **df** | **χ^2^** | **Δdf** | ***p*** | **AIC** |
| --- | --- | --- | --- | --- | --- | --- | --- |
| **Negative affect^1^** |  | 5237.88 | 2315 |  |  |  | 607.88 |
|  | **b_au_** | 5247.18 | 2316 | 9.30 | 1 | .002 | 615.18 |
| **Anxiety^2^** |  | 5244.89 | 2313 |  |  |  | 618.89 |
|  | **b_au_+b_ac_** | 5249.45 | 2315 | 4.55 | 2 | .103 | 619.45 |
|  | **b_eu_** | 5254.03 | 2314 | 9.14 | 1 | .003 | 626.03 |

^1^ α = .05, ^2^ α = .025. Subscript au stands for genetic effects unique to the psychopathology variable, eu stands for environmental effects that are unshared between twins and unique to the psychopathology variable, and ac stands for genetic effects that are common to both intelligence and psychopathology.

*Supplementary Table 9.*

Results of bivariate twin moderation model fitting: Tests whether psychopathology moderates genetic and environmental factors of intelligence.

| **Moderator** | **Test** | **-2LL** | **df** | **χ^2^** | **Δdf** | ***p*** | **AIC** |
| --- | --- | --- | --- | --- | --- | --- | --- |
| **Negative affect** |  | 4982.46 | 2156 |  |  |  | 670.46 |
|  | **b_au_** | 4982.53 | 2157 | 0.08 | 1 | .782 | 668.53 |
|  | **b_cu_** | 4982.63 | 2157 | 0.17 | 1 | .679 | 668.63 |
|  | **b_eu_** | 4982.97 | 2157 | 0.51 | 1 | .475 | 668.97 |
| **Anxiety** |  | 4985.51 | 2155 |  |  |  | 675.51 |
|  | **b_au_+b_ac_** | 4985.51 | 2157 | -0.01 | 2 | >.999 | 671.51 |
|  | **b_cu_** | 4985.52 | 2156 | <0.01 | 1 | .951 | 673.52 |
|  | **b_eu_** | 4986.56 | 2156 | 1.04 | 1 | .301 | 674.56 |
| **ODD** |  | 5303.82 | 2156 |  |  |  | 991.82 |
|  | **b_au_** | 5305.61 | 2157 | 1.79 | 1 | .181 | 991.61 |
|  | **b_cu_** | 5305.02 | 2157 | 1.20 | 1 | .273 | 991.02 |
|  | **b_eu_** | 5308.91 | 2157 | 5.09 | 1 | .024 | 994.91 |
| **Autism** |  | 4926.46 | 2153 |  |  |  | 620.45 |
|  | **b_au_+b_ac_** | 4929.86 | 2155 | 3.41 | 2 | .182 | 619.86 |
|  | **b_cu_** | 4927.05 | 2154 | 0.60 | 1 | .439 | 619.05 |
|  | **b_eu_** | 4926.49 | 2154 | 0.04 | 1 | .851 | 618.49 |
| **ADHD** |  | 5264.34 | 2153 |  |  |  | 958.34 |
|  | **b_au_+b_ac_** | 5264.56 | 2155 | 0.22 | 2 | .895 | 954.56 |
|  | **b_cu_** | 5264.66 | 2154 | 0.33 | 1 | .567 | 956.66 |
|  | **b_eu_** | 5266.94 | 2154 | 2.60 | 1 | .107 | 958.94 |

α = .017. -2LL is the -2 loglikelihood, χ2 is the -2LL ratio test
