# Supplementary material for: Are Genetic and Environmental Risk Factors for Psychopathology Amplified in Children with Below-Average Intelligence? A Population-Based Twin Study
Source: Behav Genet. 2024 Feb 14;54(3):278–89. doi: 10.1007/s10519-023-10174-7 (PMC11032279; doi:10.1007/s10519-023-10174-7)
Supplement: Supplementary file 2 — Supplementary file2 (DOCX 42 kb) [file 10519_2023_10174_MOESM2_ESM.docx]

**Supplementary Figures**

Are genetic and environmental risk factors for psychopathology amplified in children with below-average intelligence? A population-based twin study.

Behavior Genetics

Susanne Bruins^1,2^, Elsje van Bergen^1,2,3^, Maurits W. Masselink^4,5,6^, Stefania A. Barzeva^4,6^

Catharina A. Hartman^6^, Roy Otten^4^, Nanda N.J. Rommelse^6,7^,

Conor V. Dolan^1^ & Dorret I. Boomsma^1,8^

^1^ Department of Biological Psychology, Vrije Universiteit Amsterdam, the Netherlands
^2^ Amsterdam Public Health Research Institute, Amsterdam, The Netherlands
^3^ Research Institute LEARN!, Vrije Universiteit Amsterdam
^4^ Radboud University, Behavioural Science Institute, Nijmegen, The Netherlands
^5^ Radboud University Medical Center, Department of Psychiatry, Nijmegen, The Netherlands
^6^ Department of Psychiatry, University of Groningen, University Medical Center Groningen, Groningen, The Netherlands
^7^ Karakter Child and Adolescent Psychiatry University Center, Nijmegen, The Netherlands
^8^Amsterdam Research and Development (AR&D) Research Institute, Amsterdam, The Netherlands

Corresponding Author: Susanne Bruins, s.bruins@vu.nl

**Supplementary Figure 1** Histograms of the six variables. Psychopathology variables are scaled with IRT.
