# Supplementary material for: Are Genetic and Environmental Risk Factors for Psychopathology Amplified in Children with Below-Average Intelligence? A Population-Based Twin Study
Source: Behav Genet. 2024 Feb 14;54(3):278–89. doi: 10.1007/s10519-023-10174-7 (PMC11032279; doi:10.1007/s10519-023-10174-7)
Supplement: Supplementary file 3 — Supplementary file3 (PDF 1145 kb) [file 10519_2023_10174_MOESM3_ESM.pdf]

**Supplementary Methods**

# Are genetic and environmental risk factors for psychopathology amplified in children with below-average intelligence? A population-based twin study.

Behavior Genetics

Susanne Bruins[1,2], Elsje van Bergen[1,2,3], Maurits W. Masselink[4,5,6], Stefania A. Barzeva[4,6]
Catharina A. Hartman[6], Roy Otten[4], Nanda N.J. Rommelse[6,7],
Conor V. Dolan[1] & Dorret I. Boomsma[1,8]

[1] Department of Biological Psychology, Vrije Universiteit Amsterdam, the Netherlands
[2] Amsterdam Public Health Research Institute, Amsterdam, The Netherlands
[3] Research Institute LEARN!, Vrije Universiteit Amsterdam
[4] Radboud University, Behavioural Science Institute, Nijmegen, The Netherlands
[5] Radboud University Medical Center, Department of Psychiatry, Nijmegen, The Netherlands
[6] Department of Psychiatry, University of Groningen, University Medical Center Groningen, Groningen, The Netherlands
[7] Karakter Child and Adolescent Psychiatry University Center, Nijmegen, The Netherlands
[8]Amsterdam Research and Development (AR&D) Research Institute, Amsterdam, The Netherlands

Corresponding Author: Susanne Bruins, s.bruins@vu.nl

## The classical twin design

In the classical twin design, variance of phenotypes can be decomposed into additive genetic (A) and non-additive or dominance (D) variance components, and common or shared (C) and unshared (E) environmental variance components. Phenotypic differences between monozygotic (MZ) twins, who are genetically identical, are attributable to E, and differences between dizygotic (DZ) twins, who on average share 50% of their alleles, are attributable to A, D, and E. In the twin design, it is not possible to simultaneously estimate the contributions of A, C, D, and E to the phenotypic variance. Thus, the ACE and ADE models were separately fitted to the data using the largest datasets, i.e. (including all 13,542 twin pairs). The choice to use an ACE or ADE model is based on the twin correlations: $r_{mz}>2r_{dz}$ suggests an ADE model; $r_{mz}<2r_{dz}$ suggests an ACE model, and $r_{mz}\approx 2r_{dz}$ suggest the absence of D or C effects, resulting in an AE model (Plomin et al., 2013). One can test for the significance of C or D in the respective ACE or ADE models using loglikelihood ratio tests. The ACE or ADE twin model can be specified as a structural equation model and represented as a path diagram. A path diagram of the ACE twin model is presented in **Fig. A**.

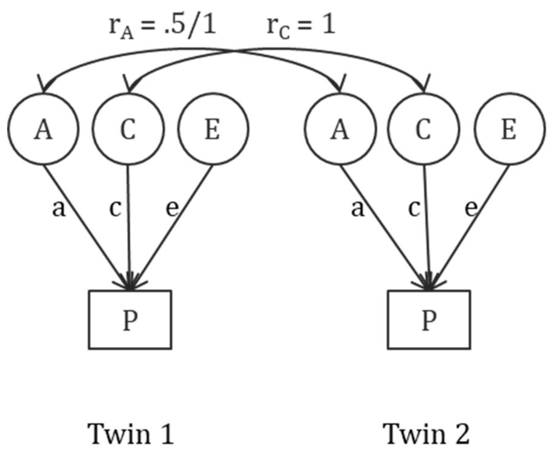


**Fig. A** An ACE twin model. Squares denote observed variables (Phenotype P in twin 1 and twin 2), circles denote latent variables. The parameter $r_A$ is the additive genetic correlations between members of a twin pair, i.e., $r_A$=1 for MZ twin pairs, and $r_A$=.5 for DZ twin pairs. Variance due to A, C, and E can be found by path tracing: $\sigma_A^2 = a^2$; $\sigma_C^2 = c^2$; and $\sigma_E^2 = e^2$

In the univariate analysis, we specify the model as: P = m + A + C + E or P = m + A + D + E, where P is the phenotype with mean m, and A, C, D, and E are zero-mean latent variables as defined above (given that we estimate the latent variances). Given the standard assumptions of the classical twin design (see Plomin et al., 2013), phenotypic variance $\sigma_P^2$ is decomposed as follows: $\sigma_P^2 = a^2*\sigma_A^2 + c^2*\sigma_C^2 + e^2*\sigma_E^2 = a^2 + c^2 + e^2$ in the ACE model, or $\sigma_P^2 = a^2*\sigma_A^2 + d^2*\sigma_D^2 + e^2*\sigma_E^2$ in the ADE model. The MZ and DZ covariance matrices in the full ACDE model are modeled as follows:

$$\Sigma_{mz} = \begin{pmatrix} \sigma_{mz}^2 & \sigma_{mz1,mz2} \\ \sigma_{mz1,mz2} & \sigma_{mz}^2 \end{pmatrix} = \begin{pmatrix} \sigma_A^2+\sigma_C^2+\sigma_D^2+\sigma_E^2 & \sigma_A^2+\sigma_C^2+\sigma_D^2 \\ \sigma_A^2+\sigma_C^2+\sigma_D^2 & \sigma_A^2+\sigma_C^2+\sigma_D^2+\sigma_E^2 \end{pmatrix}$$

$$\Sigma_{dz} = \begin{pmatrix} \sigma_{dz}^2 & \sigma_{dz1,dz2} \\ \sigma_{dz1,dz2} & \sigma_{dz}^2 \end{pmatrix} = \begin{pmatrix} \sigma_A^2+\sigma_C^2+\sigma_D^2+\sigma_E^2 & \frac{1}{2}\sigma_A^2+\sigma_C^2+\frac{1}{4}\sigma_D^2 \\ \frac{1}{2}\sigma_A^2+\sigma_C^2+\frac{1}{4}\sigma_D^2 & \sigma_A^2+\sigma_C^2+\sigma_D^2+\sigma_E^2 \end{pmatrix}$$

However, as mentioned above, based on the twin correlations, either $\sigma_D^2$ or $\sigma_C^2$ is fixed to zero to identify the model. The MZ phenotypic covariance equals $\sigma_A^2 + \sigma_C^2$ or $\sigma_A^2 + \sigma_D^2$. In DZ twins, this equals $½\sigma_A^2 + \sigma_C^2$ or $½\sigma_A^2 + ¼\sigma_D^2$ (for a derivation of the coefficients ½ and ¼, see Mather and Jinks 1977; Falconer and Mackay 1996).

**Bivariate analyses: Decomposition of (co)variance and moderation**

The bivariate extension of the twin model involves the simultaneous analysis of two phenotypes. We present the ACE version of this model. Figure B depicts the bivariate ACE model for intelligence and a given psychopathology scale. The latent A (Ac, Au), C (Cc, Cu), and E (Ec, Eu) are standardized (zero mean, variance one). The variances of the two traits and their covariance are decomposed into additive genetic components, shared environmental components, and unique environmental components. That is, in the path diagram (Figure B), the variance of intelligence equals $a_{CA}^2 + c_{CA}^2 + e_{CA}^2$, the variance of psychopathology equals $a_c^2 + a_u^2 + c_c^2 + c_u^2 + e_c^2 + e_u^2$, and the covariance between the two phenotypes equals $a_{CA} \times a_c + c_{CA} \times c_c + e_{CA} \times e_c$. The parameters $a_u$, $c_u$, and $e_u$ indicate additive genetic, shared environmental, and unshared environmental effects unique to psychopathology; the parameters $e_m$, $c_m$, and $e_m$ indicate additive genetic, shared environmental, and unshared environmental effects unique to intelligence; and the parameters $a_c$, $c_c$, and $e_c$ indicate additive genetic, shared environmental, and unshared environmental effects that are shared between psychopathology and intelligence. Note that unshared environmental effects do not contribute to the covariance of the twins (see $\Sigma_{mz}$ and $\Sigma_{dz}$ above), but may contribute to the covariance of the phenotypes within persons ($e_{CA} \times e_c$). Similarly, common environmental effects, if present, contribute to the

covariance of the twins, but may (parameter $c_c \neq 0$), or may not ($c_c=0$) contribute to the covariance of the phenotypes within persons.

The moderation model, as depicted in **Fig. C** (Purcell 2002; Van der Sluis et al. 2012), can be viewed as an extension of the bivariate model. That is, we tested if the effects of the genetic and environmental factors on psychopathology are moderated by intelligence. In Figure C, the variance of a psychopathology phenotype equals $(a_c + b_{ac} \times CA)^2 + (a_u + b_{au} \times CA)^2 + (c_c + b_{cc} \times CA)^2 + (c_u + b_{cu} \times CA)^2 + (e_c + b_{ec} \times CA)^2 + (e_u + b_{eu} \times CA)^2$, where $b_{ac}$, $b_{cc}$, $b_{ec}$, $b_{eu}$, $b_{cu}$, and $b_{au}$ are the moderation parameters. If all these parameters are zero, then the model in **Fig. C** is identical to the standard bivariate twin model, shown in **Fig.** . The test of moderation therefore involves the loglikelihood ratio test of these parameters.

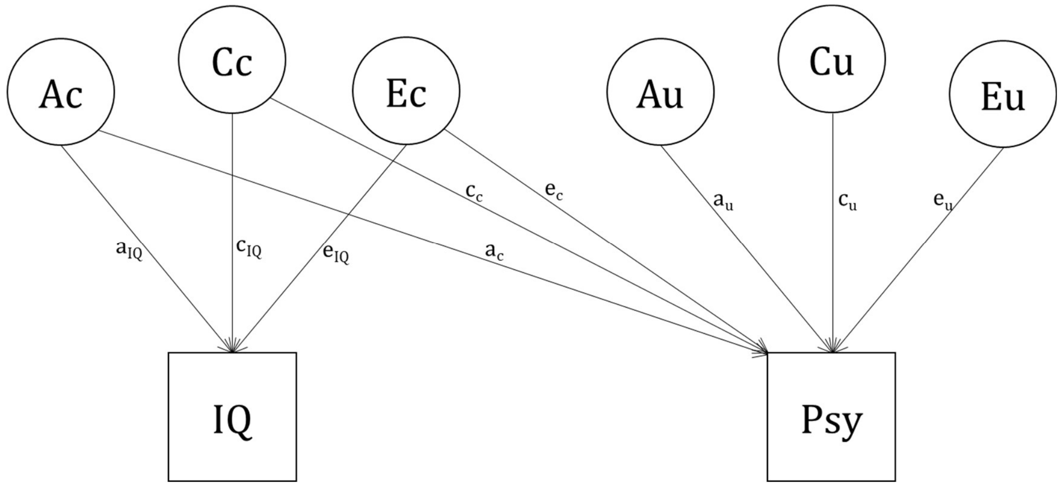


**Fig. B** Standard bivariate ACE ("Cholesky") twin model, without moderation. The moderator is IQ, and the phenotype of interest is a psychopathology variable (Psy)

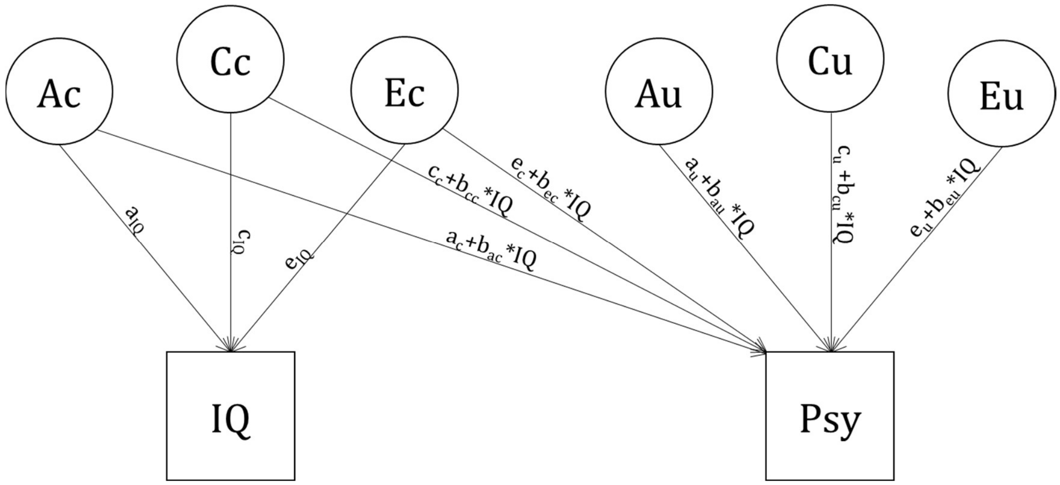


**Fig. C** Bivariate ACE moderation twin model. The moderator is IQ, and the phenotype of interest is a psychopathology variable (Psy)

**References**

Falconer DS, Mackay TF (1996) Introduction to quantitative genetics, 4th ed. Prentice Hall, Harlow, England

Mather K, Jinks JL (1977) Introduction to biometrical genetics. Chapman and Hall London

Purcell S (2002) Variance components models for gene–environment interaction in twin analysis. Twin Research and Human Genetics 5:554–571

Van der Sluis S, Posthuma D, Dolan CV (2012) A note on false positives and power in GxE modelling of twin data. Behavior genetics 42:170–186
